# Perimenopausal women’s voices: How does their period at the end of reproductive life affect wellbeing?

**DOI:** 10.1177/20533691231216162

**Published:** 2023-11-20

**Authors:** Elizabeth Ray, Jacqueline A Maybin, Joyce C Harper

**Affiliations:** 1 UCL EGA Institute for Women's Health, 4919 University College London, London, UK; 2 Centre for Reproductive Health, Institute for Regeneration and Repair, 215746 University of Edinburgh, Edinburgh, UK

**Keywords:** Perimenopause, period, menstruation, heavy, wellbeing, unpredictable

## Abstract

**Objective:**

To explore perimenopausal women’s feelings towards their periods, the impact on their wellbeing and how we can support them.

**Study design:**

Participants were recruited for focus groups through social media advertisements. In 6 online focus groups, 31 perimenopausal women aged 40–55 living in the UK were asked 5 questions relating to periods and perimenopause, support and education.

**Main outcome measures:**

Content analysis.

**Results:**

When asked How do you feel about having a period? The participants often related back to the stress of menarche and said their period negatively impacted their wellbeing their whole lives. During perimenopause, their periods had become unpredictable, heavy, and a debilitating disruption to their lives. The women desired support at work through policy, and from family and friends. They believed that educating girls and boys during school was the best place to start, but that education through work and community groups was also needed. They felt healthcare professionals should have mandatory training regarding women’s health issues and menopause.

**Conclusion:**

The study highlights the impact of menstrual experiences on the wellbeing of perimenopausal women, emphasising the need for improved menstrual education from an early age. Comprehensive menstrual education in schools involving both genders to enable informed decision-making and improved training for healthcare professionals are recommended. Support networks for perimenopausal women will promote better quality of life for women as they go through their perimenopause journey.

## Introduction

Most women experience menstrual cycles between menarche and menopause.^1^ Menarche begins around 12 years old for girls in the UK, but can start between 8 and 17 years^2^ Menstruation is cyclic shedding of the uterine lining that and occurs approximately every 24–38 days and lasts up to 8 days, though both menstrual cycle and bleeding length can vary from month to month and between individuals.^3^ The menstrual cycle begins on the first day of menstrual bleeding and lasts until the day before the next period begins.^2^ Menopause is defined as a year after the final menstrual period (FMP).^4,5^ On average, menopause occurs between ages 45 and 55, with the UK’s average being 51 years.^6,7^ After menopause, a woman is postmenopausal.

Some women’s quality of life (QoL) and wellbeing are significantly impacted by their period. Often, the initial episode of menses shapes perception and management throughout their lives – many have negative experiences around their menstruation.^8,9^

Note, in this paper we will use the term women, as the focus groups included only cis women. A separate study needs to be done with trans, non-binary and gender diverse people.

### Normal and abnormal periods

Normal uterine bleeding includes menstrual bleeding that occurs at regular, predictable intervals, and does not excessively interfere with her physical, social or emotional QoL.^3^ Abnormal uterine bleeding (AUB) affects 3%–30% of reproductive age women worldwide and is characterised by bleeding that is prolonged, irregular, frequent/infrequent, and/or heavy.^10^ AUB can occur for many reasons, such as fibroids, adenomyosis, coagulopathy or iatrogenic causes.^10^ Some symptoms that accompany AUB are pain, dysmenorrhea (painful periods), anxiety, depression and fatigue.^11^ Other reproductive tract issues, such as endometriosis, premenstrual syndrome (PMS) or premenstrual dysphoric disorder (PMDD) cause other non-bleeding symptoms, with the latter two occurring prior to menstruation.^10,11^

Heavy menstrual bleeding (HMB), a type of AUB, is excessive menstrual blood loss interfering with physical, social or emotional QoL.^3^ In the UK, HMB accounts for approximately 20% of gynaecological referrals^12^ and may be underdiagnosed due to social taboos. HMB can cause iron deficiency anaemia, resulting in fatigue and shortness of breath.^13^

Dysmenorrhea is pain that accompanies menstruation, usually located in the lower abdomen.^14^ It is one of the most common menstrual symptoms, with estimated prevalence around 45%–95% of menstruating women.^15^ The prevalence of dysmenorrhea is likely highly underestimated, as many women consider period pain normal and therefore do not seek medical help.^15^ Dysmenorrhea can have debilitating effects on women’s QoL, such as impacting social relationships, physical activities, and causing women and girls to miss work and school.^15^

### Perimenopause

Perimenopause is the time before the FMP when hormonal changes may lead to a myriad of symptoms, often beginning with menstrual irregularity. It is normal for women to experience changes in their menstrual cycle at this time, eventually leading to amenorrhea. These changes are categorised into early and late transition stages.^5^

The perimenopause experience varies among women, with estimates that 80%–85% of women in the UK have symptoms, yet only 10% seek medical care.^16–18^ Misconceptions and lack of education regarding perimenopause and menopause contributes to women being unaware that symptoms are related to perimenopause.^19^ Besides changes in the menstrual cycle, symptoms are categorised as vasomotor, genitourinary symptoms and psychological.^19–21^

Vasomotor symptoms affect up to 80% of perimenopausal women,^22^ and include hot flushes and night sweats, which are sudden, intense feelings of warmth. These symptoms often cause sleep disruptions, fatigue and decreased QoL.^23^ This can lead to lower productivity at work, as well as increased incidence of cardiovascular disease and depression.^21,24–26^

Genitourinary symptoms include genital symptoms (dryness, burning and irritation), sexual symptoms (sexual discomfort or pain, impaired function) and urinary symptoms (urgency, incontinence and recurrent urinary tract infections).^20,27^ They can arise during perimenopause, which impacts sexual function and can be extremely distressing. Decreased sexual function is reportedly notable approximately 20 months before the FMP, with the greatest decrease occurring later in perimenopause.^28^

Adverse mood changes, such as depression and anxiety, have been reported by perimenopausal women,^29^ but other factors may be the cause. A review of studies determined symptoms ‘tend to ramp up overall as women enter the late reproductive years and progress through’ early perimenopause, with the largest increase occurring towards the end of perimenopause.^5^

Physical and psychological symptoms, whether experienced singularly or in combination, can be distressing and negatively impact women’s wellbeing.

There are ways to manage symptoms associated with perimenopause. Treatments can include lifestyle changes, hormone replacement therapy (HRT) (which should more accurately be referred to as hormone therapy (HT)), other medications or alternative/natural treatments. Lifestyle adjustments include physical activity, improvements in sleep and nutritional food choices, which have the added benefit of mitigating the risk of osteoporosis, and other disorders such as heart disease and dementia.^30–32^ Cognitive behaviour therapy (CBT) has been shown to be beneficial for low mood and anxiety.^33^ Additionally, vaginal estrogen in the form of a vaginal tablet, pessary, cream, gel or vaginal ring combats vaginal dryness.

### Menstrual and perimenopause stigma

Stigma is defined as a mark of disgrace associated with particular circumstances or qualities that sets a person or group apart.^34^ Menstrual stigma exists globally.^35^ Despite periods being a common, natural occurrence for half the population, some cultures view menstruation as a dirty, shameful occurrence. These misconceptions have led to women around the world being banned from some physical spaces (such as places of worship), forced isolation, and restrictions on activities such as bathing and food preparation.^36^

Stigma, harmful policies and poor working conditions impact education and economic opportunities for many girls and women, potentially leading to early marriage and reduced employability.^36^ In the UK, over a quarter of 16–24-year-old women report experiencing period shaming.^37^ Additionally, approximately 6 million women avoid exercise and 2 million miss work due to periods.^37^

Stereotypes in popular culture reinforce negative perceptions of stereotypical physical, emotional and psychological symptoms.^38^ Public Health England, through surveys and focus groups, reported that women experienced shame, embarrassment and discomfort surrounding reproductive health, which in turn influenced symptom management and decisions to seek care.^39^ Social expectations, attitudes from GPs (general practitioners), and perceptions at home, work and school contribute to these feelings.^39^

Between the UK government’s Women’s Health Strategy, the Scottish Government’s Women’s Health Plan, the British Standards Institution (BSI) Guidelines, and the development of a UK menopause education and support program, work is being done across the UK to improve conditions around perimenopause.

While women have been asked about knowledge and attitudes toward peri/menopause, to our knowledge this is the first study to specifically solicit perimenopausal women’s attitudes towards their periods and the effects on their wellbeing. Additionally, the study aims to understand how women envision improvements to education around menstruation and perimenopause. The lack of obtaining women’s opinions in these areas is a notable gap in research.

## Methods

### Ethics

Ethical approval was obtained from University College London’s (UCL) ethics committee (reference 9831/008).

### Recruitment and data collection

Recruitment was achieved through distribution of a recruitment poster that was shared online via Professor Joyce Harper’s social media accounts on Twitter, Instagram, Facebook and LinkedIn. Additionally, the study details were shared through personal and professional contacts and word of mouth. Recruitment went live on 17th May 2023 until 25^th^ June 2023. Inclusion criteria for selected participants were women living in the UK, who had symptoms of perimenopause, and were still experiencing periods at the time of entering the study. As most women experience their FMP between 45 and 55, we included an age range of 40–55.

Written, informed consent was obtained from all participants, alongside the study information leaflet. Participants were sent a zoom link and further details of the questions and focus group format. All participants were given a pseudonym which was used on the zoom call to ensure anonymity. For added anonymity, the pseudonyms were replaced with a number for this publication. Participants consented to their anonymised data being included in the scientific literature and were aware that anonymous quotes may be used on social media, in presentations and in a book. After the focus group, they were given a £25 voucher as compensation for their time.

Over 100 women reached out to be a part of the study. 41 women met the criteria and were invited to a focus group session. 10 women that were invited either did not show up to their session or were removed from the sessions due to not turning on their video to confirm they were the correct participant prior to recording, which is explained in depth in the discussion under limitations.

The focus groups consisted of five questions: the first three focused on women’s feelings towards periods, changes during perimenopause and the impacts on their wellbeing.

(1) How do you feel about having a period?

(2) Has your period changed during the perimenopause?

(3) How does your period affect your wellbeing?

The final two questions enabled the team to understand how to better support and educate everyone on menstruation and perimenopause.

(4) What support do you need to manage your menstrual cycle/periods during the perimenopause?

(5) How could we improve education around menstruation and perimenopause?

Once participants joined the Zoom session, the researchers ensured their display name matched their randomly assigned pseudonym. The focus groups were run from May 31–June 29, 2023. Interviews lasted between 60 and 85 min. Participants were monitored for signs of distress, including withdrawal, visibly upset/crying, shaking and tremors in speech. Contact details for the research leads, as well as information for counselling and mental health services were on hand in the event participants wished to discuss anything further or desired more professional assistance, though they were not needed throughout the study.

The Zoom video recording was immediately deleted. Then, the Zoom transcript was downloaded after each focus group, checked for accuracy with the audio recording, and deleted.

The number of focus groups aimed for were between 5 and 10, with the final number decided when it felt that saturation was reached, that is, the last focus group did not provide any new topics. In total, 6 focus group sessions were held consisting of 3–7 women per group, totalling 31 women interviewed.

### Content analysis

Immediately following each focus group, the team discussed the main findings from the group. A summary of each session was written to accurately encapsulate the discussion and tone and begin understanding initial thoughts and ideas. After completion of all 6 focus groups, each session’s transcript was read closely to ensure familiarisation. Transcripts were then broken apart by question, allowing answers to each question to be read in full, and was then analysed individually to identify initial codes. NVivo (version 14) software was used to code transcripts. By using notes gathered during familiarisation based on answers to each question, initial codes were systematically distinguished, and relevant data was sorted into each code. This allowed for further familiarisation with the data, as well as a chance to better recognise patterns. Repeated thoughts and particularly meaningful sentiments were noted alongside initial thoughts and ideas pertaining to the data. Codes were then sorted into themes that were reviewed against the overall dataset to ensure that it was representative. Themes were refined iteratively by both researchers until it was determined that the overall narrative of the women was adequately conveyed.

## Results

### Demographics

In total, 31 women took part in the focus group sessions (Table 1). The average age of the participants was 46.9 years.

**Table 1.** Participant Demographics. Demographics and symptoms of the perimenopausal women in the study.

Reference	Age	Ethnicity	Symptoms
1	48	Any other White background (please specify) - Spanish	Discomfort breast, heavy legs, brain fog?
2	43	White - English/Welsh/Scottish/Northern Irish/British	Brain fog, trouble concentrating, mood changes
3	42	White - English/Welsh/Scottish/Northern Irish/British	Mood changes. Night sweat
4	47	White - English/Welsh/Scottish/Northern Irish/British	Brain fog, low mood, sweats, heart palpitations
5	48	White - English/Welsh/Scottish/Northern Irish/British	I have been on HRT for around 15 months, so my symptoms are more managed. I endured symptoms I think for around 3 years before I started to suspect that something wasn’t right. I hadn’t even heard of perimenopause until I did some online research into irregular periods. I didn’t think it was my time though. However my symptoms progressed, in particular brain fog and my emotional state (I felt suicidal at one point, and confidentially I did have to leave my last job due because I didn’t report my perimenopause symptoms (my performance was deemed to be inadequate and I had no choice to but leave). Although I am on HRT I still find that I suffer from brain fog, anxiety, fatigue (I do question whether I need to review my prescription now!) My periods are irregular. Sometimes they are really light, sometimes really heavy, maybe I will have a heavy period which will last for 2 weeks, then a really light one 2 weeks later that lasts 3 days. I also find that my period isn’t affected by HRT (micronized progesterone), the cycle of HRT doesn’t follow the cycle of my periods. I never have huge gaps between my periods, I do bleed on a monthly basis (just for varies lengths of time). Thankfully I don’t suffer any pain. I used to pre-children but since then I have been pain free. It was only stomach cramping, nothing more. Hope that all helps!
6	47	White - English/Welsh/Scottish/Northern Irish/British	Great variance in my periods in terms of frequency and flow. I’ve rarely had painful periods but in the last year I have had some that have debilitated me, leaving me unable to focus on my work or other tasks for 1–2 days
7	45	White - English/Welsh/Scottish/Northern Irish/British	Menstrual irregularity
8	46	White - English/Welsh/Scottish/Northern Irish/British	Heat flushes
9	51	Asian/Asian British - Pakistani	Loss of strength
10	48	White - English/Welsh/Scottish/Northern Irish/British	Digestive changes and discomfort including bloating and increase frequency of defecation; increased severity of PMS symptoms; increased body odour; mood swings; hot flushes; mild bladder incontinence; occasional anxiety; disrupted sleep
11	42	White - English/Welsh/Scottish/Northern Irish/British	Changes in sexual desire, headaches, vaginal dryness
12	50	White - English/Welsh/Scottish/Northern Irish/British	Changing cholesterol levels, changes in sexual function, etc.
13	45	White - English/Welsh/Scottish/Northern Irish/British Asian/Asian British - Indian	Menstrual irregularity
14	43	White - English/Welsh/Scottish/Northern Irish/British White - Irish	Mood changes, heat flushes
15	47	White - English/Welsh/Scottish/Northern Irish/British	Irregularities of menstrual period and hot flushest
16	44	White - English/Welsh/Scottish/Northern Irish/British White - Irish	Mood changes, heat flushes
17	51	Asian/Asian British - Pakistani	Brain fog, tiredness, lack of sleep, distressing emotions
18	47	White - English/Welsh/Scottish/Northern Irish/British	Irregular period, mood changes
19	49	White - Irish	Brain fog, fatigue, menstrual migraines, problems concentrating, dizziness, skin problems; I do also have M.E. and there is a certain amount of crossover involved with these symptoms. Difficulty sleeping, mood swings
20	46	White - English/Welsh/Scottish/Northern Irish/British	Changes in sexual function, changing cholesterol levels
21	47	Any other Asian background - Sri Lankan	hot flashes, brain fog, fatigue, urge to urinate, anger, blurred vision
22	50	White - English/Welsh/Scottish/Northern Irish/British	Night sweats, memory issues/brain fog, irregular periods, mood swings
23	40	N/A	Vaginal and bladder problems
24	48	Asian/Asian British - Pakistani	I experience a wide variety don't know if they are the same in other women. Irregular periods, hot flashes, night sweats my bed becomes so wet, mood changes and I feel so bad after this, sleep disturbances, vaginal dryness which has caused lots of worry, decreased libido
25	44	Any other White background (please specify) – British/American/Italian	Mood swings, erratic periods, low libido, weight gain
26	54	White - English/Welsh/Scottish/Northern Irish/British	Flooding, phases of irregular periods, irritability, brain fog, palpitations, increased digestive sensitivity, thumb/wrist joint pain, occasional hot and cold flushes
27	49	White - English/Welsh/Scottish/Northern Irish/British	Night sweats, low energy, brain fog, trouble sleeping
28	47	Any other white background - Canadian	Weight gain, change in periods (shorter, more intense), low libido, breast swelling/tenderness (what is happening to my freaking nipples?!) and finally bit of vaginal dryness. So much fun!
29	48	White - English/Welsh/Scottish/Northern Irish/British	They are petering out and barely last a day now, but I haven’t gone a month without anything yet. I still get PMT though. I’m on HRT now, so sorry if that excludes me. However, I was extremely anxious and on such a short fuse, I felt wound like a spring. I had the occasional night sweat, constant, comedy brain fog, my libido was on the floor and a lack of motivation. PLus, my periods were hit and miss. Some months I had one, others not, where before I was regular as clockwork
30	52	White - English/Welsh/Scottish/Northern Irish/British	Changes to periods, brain fog, joint pain, insomnia, fatigue, dry eyes, digestive issues, low mood, tearful, poor concentration, migraines, heart palpitations, night sweats, thinning hair, weight gain
31	45	White - English/Welsh/Scottish/Northern Irish/British	Hot flushes, mood swings, insomnia

### Content analysis: The good, the bad and the bloody

The analysis was broken down by research question. Themes and subthemes were identified for each question. The women were eager to tell their stories, to be heard, and to voice their ideas and suggestions on what needs to be done to improve menstrual support and education. A few women reached out after their session to thank us for our research and expressed their gratitude at having been given the chance to tell their stories – especially if it could help other women.

### Question 1: How do you feel about having a period?

Each woman had a unique, individual experience with her period. While many of the participants opened with strong, negative feelings towards their periods and associated symptoms, some described some positives. Of those who did have a more positive outlook, they still struggled with the negatives that come alongside menstruation. A lot of women expressed mixed feelings, oscillating between the good and the bad while telling their stories.

From this question, 4 themes were identified: negative feelings from puberty to adulthood, mixed feelings, women without children and experiencing lack of support from healthcare professionals.

### Negative feelings from puberty to adulthood

Every participant recounted at least one negative period experience over the course of their life. Although each person had a unique story, they all shared negative sentiments. Negative feelings fell into two subthemes: puberty and negativity as an adult.

#### Puberty

Upon asking the women how they felt about their periods, most of them reverted to memories of puberty, as if it had stayed with them throughout their lives. As they reflected, many reported what age it began, whether they had anticipated it or were caught unaware because they had not learned about periods, and whether they had started menstruating earlier or later than what they perceived to be a normal age. For many of the women, negative feelings towards menstruating blossomed with puberty.

*“It was the bane of my life, quite honestly. So, I started when I was 12. I've always had a 23-day cycle, and whenever I told anybody that they always said, ‘No, no, no, you have to count,’ and they were telling me how to count like I couldn't count, because apparently that's not possible.” [#17]*

*“I got my period for the first time on an aeroplane, by myself. It was the first time I was traveling, I must have been just under 12.…And I was just like, ‘okay, so either I'm dying or…this is like bad.’ My mom is all like, ‘Yeah, you're a woman.’ I was like, ‘Oh, cool…’ that sort of just made me like, if this is what it is to be a woman - that I just bleed for 7 [days]…I feel like there's maybe other things that we could do to be a woman. I hate it. I don't like the way it smells. I don't like tampons. I don't like any of it. And it’s just yucky.” [#28]*

One woman said she hated them, citing ‘incredibly heavy’ periods that soaked through her underwear during school.

*“I hate them…I was 13 when I got my period, and they were incredibly heavy - really heavy. Soak-through-your-underwear kind of heavy. Really awful at school kind of thing…” [#21]*

One woman said her period has been ‘stressful and traumatic’ and recalled her first period had started in school, which she felt was embarrassing.

*“I feel it's one of the most stressful and traumatic parts of my life. I had a lot of experiences that were not very pleasant to me, especially when I was still in school when it started…And my parents didn't really talk about that then, so when it happened, I was young. And it happened right in school. It was so embarrassing for me.” [#16]*

#### Negativity as an adult

Women went on to say how periods had affected their QoL as an adult. Negative comments included frustration, stress, inconvenience, messiness, anxiety and dread towards their period. The reasons for these feelings ranged from seeing it as a ‘waste of time’ after never having had children, to being fed up and stressed over the heaviness, to the frustration of it coming at an inconvenient time. One woman detailed just how terrible her periods were, with symptoms so debilitating that she would be out of work for days at a time, leaving her feeling constantly drained of energy. Her life, from menarche onward, was at the mercy of her menstrual cycle.

*“I couldn't work for 4 or 5 days at a time. My migraines were so intense…to the point of the fact that I'd lift the duvet and I’d vomit because of the touch...and also before my period, I was just so down and upset. During my period, I was just so angry I’d kill anybody. After my period, I was just so washed out, and then the next cycle would start. It was literally a week of each of this, and that was my life…” [#17]*

A woman who experienced endometriosis-related symptoms throughout her life but was not diagnosed until trying to start a family, recounted that she used to have terrible periods.

*“…a female colleague when I was temping…this woman was I think, 10 years older than me, and I think I'd been off, or I'd been vomiting somewhere or something. And she was like, ‘Oh, my God is this a regular thing?’ And I said, yeah…it's like someone stabbing me, I vomit, I faint…And that was that and it was completely missed.” [#4]*

While trying to conceive, she not only experienced her usual period pain, but also the compounded disappointment and heartbreak of not being able to conceive prior to trying IVF.

*“Every time I got them when I was trying to get pregnant, it was incredibly disappointing as well. But also incredibly, incredibly painful.” [#4].*

A woman with Crohn’s disease noted that her period causes her condition’s symptoms to become more intense.

*“So, whenever I get my period, like the few days before, it's like my Crohn's Disease is like, ‘I will not be shown up by this menstruation business. Let me kick off as well.’ …I spend a lot of time in the bathroom. It's not fun.” [#28]*

The woman above was not alone. Another woman also noted that her periods cause symptoms from her other conditions to worsen as well.

*“I do have a series of syndromes in my life. So, I have ME, CFS, fibromyalgia, recently told I have ADHD, and everything that comes with those. And I'm very aware of, as I get closer to bleeding, all the symptoms tend to get far worse. So, I see a cyclical pattern with generalized pain and fatigue. You know the deep fatigue, not just being tired, leading up to a period, and the antsy-ness around the ADHD, I guess, is definitely heightened.” [#30]*

Many said that periods are mainly events to get through – to put your head down until it is over.

*“Just one of those things that you have to manage, which I think is a female attitude to life is like, there's something there, we’ve just got to manage it and get on.” [#6]*

Throughout the sessions, many women echoed the above thoughts on having periods – they are something to manage and get on with.

### Mixed feelings

Some of the women expressed mixed emotions towards their periods. Throughout their reflections, they oscillated between feelings of resentment and dread, to being in awe of what their bodies are capable of. Some women saw it as an anchor to womanhood, allowing them to tap into the joy of being a woman, a way to feel grounded and connected to their body, while still acknowledging that it wrought damage both physically and emotionally.

*“I want to say I've been having mixed feelings…when it started…as a teenage girl, you're still excited, you're confused. You don't know what to do. You meet your mom. Your mom is telling you this is normal. You're checking online. And you're like, Oh, this is normal. So it's quite an exciting moment. As a teenager, you’re growing into womanhood, and you're feeling it, but then, at some point, you feel this is much stressful every month. You're having this series of hormonal changes…So it's that mixed feeling. Some months you're like this is womanhood, this is the joy of womanhood and then some of the months you’re like, ‘Why do I have to go through this stress? Why did nature have to make me undergo such stress?’” [#31].*

*“I'm…54, and they're still going strong. So, I'm a little bit resentful about them, but at the same time I do really appreciate them for what they are in terms of keeping me in tune with some kind of cycle and keeping me grounded, and and they are part of who I am and what I am.” [#26]*

*“I feel very held by my menstrual cycle, but there are these massive challenges, so… knowing that there's going to be huge amount of pain coming in a strange place, and there is going to be nausea…and feeling like I can't be in my body for however long that happens during my period, which leaves me kind of resentful because…as bleeding is going to peter out and stop…all I want is to enjoy the cycle and to enjoy my bleeding time. So, I get this little pocket of bliss and then…it will descend into something quite horrific. So, I'm wildly in love and in wild pain as well. So, it's not a clear-cut answer.” [#30].*

*“I'll be sad when they finish because it'll feel like an end of a life point, but at the same time it'll probably be quite liberating, once I get used to it.” [#5]*

Some women held an appreciation for the cyclical nature and consistency of their periods. Even through their negative experiences, some of the women chose to see the silver lining, which gave way to amazement and love towards their bodies and periods.

*“…it can sometimes feel quite inconvenient, I suppose. I've had a baby, and then during that time of being pregnant, and then breastfeeding and not having a period, it was actually quite nice…I suppose it can be quite messy…but I think that having a period is an amazing thing as well. So, it's kind of like…what our bodies do…each month.” [#2]*

One spoke of her dread towards her periods when she was quite young, but later in life decided she would benefit from changing her mindset.

*“…I had a mental switch somehow, like a light bulb moment that actually, if I started to look forward to it and see it as a sign that my body is working and doing what it should be doing, even if it is crippling me for a day, things did start to shift. I mean, I was diagnosed with PCOS, and I do fit a lot of those symptoms. And there was still a lot of that present, but certainly it was better.” [#10]*

A few women even described honouring their cycles by painting with menstrual blood or using it to feed plants.

*“I am very much about being with my menstrual cycle and I painted with blood, and so I have the love, I have the absolute love of what is possible with the cycle…So I feel very held by having a period. I feel very held by my menstrual cycle…” [#30]*

*“It's something I really enjoy, mainly because I use my menstrual blood to feed all my plants in my house and my garden…What I found is that by bleeding on the earth that really helps me to feel more connected and to honour my cycle, and I think that there was so many years that I didn't honour my cycle, and now for the last, probably 10* *years or so, I've really connected into it, and made that time to…really honour myself, and, and I've had children, and whilst it's still there, you know, it's a joy…honouring the Earth, grounding, connecting and just really honouring my cycle and that time I have to bleed, and honouring the fact that I've had children.” [#25]*

One woman, who follows the Red School teachings said she feels ‘very anchored’ because of her cycle and wonders how she will manage once it is completely gone.

*“…the good thing about it is, I don't just look at my period as the days I'm bleeding. I don't know if anyone's familiar with the Red School, but yeah, I love the Red School. And when I first learned about - because I track everything, I have the Clue app…so I feel very anchored, very anchored with my cycle. And I'm a bit... not worried, but what am I going to do once that's gone, because I plan my life and my cycle…as much as it's not really my favourite thing in the world, I've come to rely on it, and I run my life by it. And it works for me.” [#27]*

Even through negative feelings, pain, resentment and annoyance, they were still able to find comfort and amazement in their working bodies. The women exuded a sense of mixed feelings surrounding their periods, especially as they move closer towards menopause and the FMP.

One woman truly captured the attitudes towards periods, by wanting to celebrate the end.

*“I cannot wait to stop having periods. I'm gonna throw a party!” [#19]*

Once she expressed her idea of having a post-period party, many other women thought it was a wonderful, clever idea and exclaimed they would, too.

### Women without children

Some of the women never had children, and some never wanted children. Of these women, many expressed a negative sentiment towards their periods for a variety of reasons.

*“So, I come from the viewpoint of never having children. So, it has been inconvenience, or it's just one of those things that you have to manage, which I think is a female attitude to life is like, there's something there, we’ve just got to manage it and get on.” [#6]*

*“...also never had children, so always feels a bit like a waste of my time.” [#26]*

*“I like never wanted to have kids. So, I was just like, why do I have to endure this if I don't even want kids? Like somebody else should have this uterus and do something useful with it, because I am not using it. It's holding me back.” [#28]*

The woman below, who endured excruciating periods, said that she had intended on having children, but never did and now feels quite resentful that she had to endure years of pain and medical gaslighting.

*“The irony is I always really wanted children and I didn't have any, and I kind of feel really resentful of the fact that I had so many periods for nothing…” [#17]*

A few other women mentioned that they did not have any children but did not go into detail about their feelings towards it.

### Experiencing lack of support from healthcare professionals

Some of the women had serious issues with their periods and had sought medical treatment, but not everyone who sought medical attention was treated well. One woman recounted a time when she was seeking help for what she suspected was polycystic ovary syndrome, and instead found an uninviting, racist environment.

*“I went to [xx] Hospital, and I…saw the gynaecologist, English gentleman who told me…that I didn't have polycystic ovaries, I had excessive hair because…He said, “people of your ethnicity will always have”, and I'm like… I wear my eyebrows on my chin, and all here (gestures) That's not normal! He’s like “it is for your people.” Then he also told me that I was fat…I went away, lost 2 stone, came back, and he said, “Well, you're not fat, but you're still a Paki…he then proceeded to tell me that…it couldn't possibly be as bad as I described.” [#17]*

Several women felt undermined by health professionals. One woman described returning to the hospital in search of answers and that no one would believe her.

*“It was like as if it was all in my head…” [#17]*

Another woman spoke of a time when she was at a summer camp when she was younger and was noticed by a woman at the camp because of her severe menstrual symptoms of vomiting and fainting. The woman described endometriosis and suggested she should speak to a GP. She recounted visiting the GP, going to the hospital for an ultrasound scan, and being told nothing was wrong with her.

*“Oh, you've got a marvellous, wonderful, healthy womb.” [#4]*

Later when she decided she wanted children, she experienced fertility issues, which led to more testing, and finally, an endometriosis diagnosis. She was able to have a child via NHS sponsored IVF treatment but felt angry that she had to wait so long for a diagnosis when she could have instead made a plan regarding her fertility. Others experienced pain that was brushed aside by their doctors, leaving them frustrated, angry and feeling unsupported.

*“I have adenomyosis pain that…I haven't managed to have the medical world support.” [#30]*

*“It's the results of a trauma. It's a result of…not being given much choice at the time of terminating a pregnancy at 20 weeks due to anomaly. And I was left in a huge amount of pain, but I was told I wouldn't be in pain.” [#30]*

### Question 2: How has your period changed during perimenopause?

Each participant noted experiencing some degree of change to their menstrual cycle as they entered perimenopause. Between the difficult to control emotions, the unpredictability of cycle length and quantity of bleeding, they expressed anxiety, discomfort and a general sense of inconvenience to their period, which many had come to rely on and expect in a cyclic manner.

Three themes were identified for this question: unpredictability, heaviness and PMS changes.

### Unpredictability

Almost all the women spoke of having unpredictable periods that accompanied the perimenopause. Many women said their periods had followed a regular, predictable pattern for most of their lives, but since entering perimenopause, that was no longer the case. Most of the women saw changes in cycle length, period duration and the amount of blood flow. Some women experienced longer periods, while others now bled for only a few days. The constantly changing nature of their periods rendered it unpredictable in most cases.

*“And so that over these…years, I've kind of fluctuated by having the ones that are like a couple of days, to ones that are really heavy, but not like ridiculously heavy, like some women will have to deal with. But it'll just come really, really heavy and then suddenly disappear…or other months have just been really heavy for a long time…and when is this going to end?” [#5]*

Many said that the length of their cycles had changed, as well, with some saying they had become longer, whilst some had become shorter. In some cases, it seemed to come every 3 weeks, while others would have gaps of 60 or more days.

*“...sometimes I'll go 60* *days in between periods. Sometimes it's twice a month. Sometimes very light, and you think, oh, gosh, maybe this is it. Maybe it's getting lighter and lighter. And then then it totally catches me off guard not expecting it. No symptoms, not even a stomach cramp. And then whoosh and which is really kind of hard to cope with, because you're not expecting. So yeah, I'm just sort of all over the shop at the moment.” [#22]*

Whether lighter, heavier, longer or shorter, many women stated that there was no regularity: 1 month may bring any combination and the next month could switch.

***“…*** *when I walk sometimes it comes unannounced and I feel sometimes embarrassed…I'll be walking, entering some buses and be embarrassed by the stains of blood. And it does mean a lot, a lot of change during this perimenopause stage…my sleep pattern has changed. I…find it very, very hard and very difficult to sleep. I also have some irregular period. Sometimes it comes very light, scanty, less blood. Then on the next one when it comes, very heavy… And it's quite, very, very challenging. And then sometimes I have a lot of hot flashes. My body becomes hot and palpitation and I feel very, very uncomfortable and praying that this stage of my life should go…” [#24]*

Most stated that due to constant and unpredictable changes, it was challenging to commit to plans in case they coincided with a period that would physically or emotionally render them too exhausted to cope outside of their homes. In terms of coping, many found it difficult to predict the start of their period, as it no longer followed a predictable cycle. Some of the women experienced anxiety as a result of their failure to schedule and keep social engagements because they frequently filled their diaries with plans that they ultimately had to cancel.

*“But it's always been really tricky, like pain… pain the first day and heavy bleeding the second. The good news is by day three, it's almost finished. So, if I can just survive three days, you know, two days. But the problem is I have to sort of earmark my calendar, I can't plan anything. And of course, it's plus or minus two or three days. So basically, it's like a week where I can't…I'm supposed to go on a sailing trip. And it's like, well, I can't be on a sailing trip with my period.” [#27]*

### Heaviness

Many of the women talked about how heavy their periods had become with perimenopause. Some spoke of a sudden, heavy period catching them off guard and leading to embarrassing situations, such as flooding on buses or at work.

*“Then perimenopause kicked in…I didn't actually realise what was happening, but every few months I would have an incident where I literally flooded. I did it in restaurants. I did it on buses, and you know, when you were just like ‘that's not even possible,’ the sheer amount of blood. And they were always… really, really heavy…then during lockdown, I started to get 55* *day bleeds 44* *day bleeds, 22* *day bleeds and some days I wouldn't have a period for 6* *days.” [#17]*

Further, having heavier periods led to many of the women having lower iron levels, leaving them exhausted in ways they had not previously experienced.

*“Actually, it’s been irregular most of my life. Suddenly it was regular as clockwork, but recently it's becoming irregular again. So, sometimes I'm not prepared. I've booked up loads of things to do, and it can be really very disruptive, and it can exhaust me for the following week if I have to carry on with my plans. And I've got this really heavy period. So that's becoming more difficult to manage.” [#26]*

### Premenstrual symptom

Many women stated that their PMS symptoms were more intense and lasted much longer than before. This ranged from having anxiety that they had not experienced before, to uncontrollable mood swings, to generally longer periods of PMS. One woman mentioned that her PMS started lasting up to 2 weeks, which she had never experienced previously. Further, a few women stated that their PMS symptoms were ‘ramped up’, causing more emotional distress and less patience with those around them.

*“I would definitely echo…that everything, all the kind of the tension symptoms seem dialled up to 11. It's all more: more anger, more irritation, and I feel that I can cope with regular stress, but it's unnecessary stress when people do things that are just particularly unnecessary, my patience is thinner than it would have been maybe 10* *years ago. And the anxiety certainly as well.” [#19]*

*“The changing is hard. You know, the changing of not knowing when it's coming. You know that uncertainty, and then the drawn-out PMS. Which I've never had. I've always had a really good cycle, and now it's like, Oh, God! PMS! Going on for like 8* *days, or 10* *days, or 14* *days…But you know it's- I think it's something that it's almost like you get taken over by this wave of heat and anxiety and overwhelm. But it just* ***[bomb noise]*** *almost blows up. And it's- And you kind of feel like you're not you anymore.” [#25]*

### Question 3: How does your period affect your wellbeing?

By the time the women were asked specifically how their periods affected their wellbeing, most had already answered this in the first two questions and had covered how negatively their wellbeing is impacted by having periods and how they have been further impacted with the onset of perimenopausal changes. Further, while answering this question, most of the women focused on how perimenopausal changes affected their wellbeing. Two themes were identified: symptoms and lowered confidence.

### Symptoms

Though the women were not specifically asked about period symptoms, almost every single woman described their physical and emotional symptoms at one point or another. Some spoke of having intense symptoms from puberty, others described their first periods as mild or without symptoms completely. Others said they developed or changed symptoms throughout their lives – some for the better and some for the worse, especially as they entered perimenopause. Physical symptoms included cramps, sweating, breast tenderness and exhaustion. Emotional symptoms ranged from anxiety and depression to mood swings and irritability.

*“When it started, it was quite okay. I didn't have much of the symptoms.” [#3]*

*“I had serious [sic] of headache, traumatic headache. I felt very uncomfortable. My breasts became so tender. And I had a lot of mood swings. I became so anxious at any slight provocation. I was very uncomfortable.” [#24]*

*“At the early stage I didn't really have the whole cramps, or the whole mood swing thing. It was just normal…over time, it got to this point where I -started started feeling cramps. And then the mood swing thing came in. I think it's a thing called…PMS. Where I would just you know, be sad one moment, and then the next I'm like happy, and then the next is like…I don't have control over my emotions and everything. And then beside that, there was also these food craving thing where maybe this month I'm just craving one random, weird food…” [#8]*

Many of the women described the emotional roller coaster that comes with perimenopause. Many feel they have a lack of control over their moods, with some saying they experienced unexplained rage or times of sadness and sometimes lash out at family and friends, or even strangers.

*“It's frustrating. I think it's that…sometimes you just don't have the control, do you? Just kind of lash out, and then you go, God that was really embarrassing, like people in the tube, someone might touch you and you’re just like…evil look and then you kind of go, Oh, no, it's not…It's PMT, it's my period. It's embarrassing.” [#29]*

### Lowered confidence

While talking about how periods and menstruation affected their wellbeing, a few women commented on how perimenopausal symptoms and its association with getting older lowered their self-confidence. A few women reflected that they are no longer ‘young, energetic’ women, and have passed that phase of life. While some women were happy to have aged, others felt it impacted their sense of wellbeing and confidence.

*“I feel like I have reached a new phase in my life, where I'm more focused on my own health and wellbeing. Although sometimes I miss the days when I was younger and more energetic. This has changed my life very drastically; I feel sad at times knowing that I'm no longer a young woman and cannot do all the things I used to do.” [#15]*

*“The way it has affected me is that my social skills have like really gone down because…I'm seeing young girls walk around… it just dawns on me, “Okay, I've passed through this phase like…I no longer get to ovulate any longer, get to see the whole normal period thing,” and somehow it has really affected the way I-I socialise with people because…I don't know, to some extent I just tend to look down on myself. I know it's a normal adult like…adult women thing. But then it…just really doesn't give me the space to like express myself…” [#8]*

### Question 4: What support do you need to manage your menstrual cycle/periods during perimenopause?

Participants described numerous ways they need support managing their periods during perimenopause. Their needs ranged from emotional support from friends and family, to support in the workplace from colleagues and managers. Many discussed their desire for more menopause support groups. One woman described her positive experience with a menopause cafe at a previous employer. Themes identified were support in the workplace, emotional support and support groups.

### Support in the workplace

Some women worked from home, and expressed this allowed them to cope with periods (especially the unpredictability) as they did not have to deal with the anxiety of flooding while in the office or having to garner the understanding of managers.

*“I work mostly from home, so I can just… it's really easy for me to manage. The only time that's not to is also do a fair bit of teaching, so I have had days where I'm in college, and they've been like - I've had heavy clots or flooding…” [#26]*

Though a few worked from home, others emphasised the need for support in their place of work – this came out as the topic most heavily talked about in the groups. Some mentioned the need for support and sympathy from managers and colleagues, as well as mandatory managerial training. Some participants believed just knowing they had someone to talk to about any issues (perimenopause related or not) would be beneficial.

*“And you just-you just-I think people just need to know they can come and talk to you. And then likewise in my team, someone progesterone wasn't agreeing with them, so that was quite traumatic. so, I think-I guess, in the workplace it'd be nice if you're- have the mindfulness for it. If, just knowing that you can go and talk to your manager or someone about, I think, is super, super important. We have mental health first-aiders, maybe it's more part of that, not really sure.” [#29]*

*“But the emotional roller coasters of ups and downs and things I don't know, I think it can only be helpful, but there's more of an awareness with colleagues in the workplace. And I know [workplace] are creating a menopause group and menopause policies in HR and things like that. So, any kind of more understanding that you get from colleagues that maybe this is the point of life that you are, then I think that can only be a good thing.” [#22]*

Some desired more flexible working schedules that would allow them to work from home on days when they experience heavy periods or other perimenopause symptoms that are difficult to manage in the workplace.

*“So, I think from that point of view, supporting you know, like, I work set days in the office because I kind of work with another colleague, and we make sure there's one of us in every day. And sometimes I might have had, you know, might be a heavy day. And it'd be nice not to have to actually go in, be able to have the opportunity to work from home. Because, you know, because of my periods or because of some other perimenopausal symptoms that I'm experiencing.” [#21]*

One woman described how a previous educational workplace had been focused on relieving period poverty for its students, then a member of staff started a menopause cafe for teachers. In addition to having menstrual products available for students, the school also provided them in the women’s bathrooms, as well as having spare articles of clothing in the case of a surprise period or flooding.

*“Before I started at [workplace], I was a teacher, and we had a brilliant new member of staff…deputy head. She started, and she wanted to start a menopause cafe, which I hadn't really sort of heard of, I didn't know what that was. And then she sort of brought in the idea. It was a really…brilliant college, it was a Sixth Form College, and they were very much trying to tackle period poverty. So, they already had free, you know, the students could literally just help themselves to tampons, and they had everything from the kind of the cups, is it called a moon cup? The the re- the ones that you can actually reuse- I forget what those ones are- so it was brilliant. And they had a nurse. It was just a state six form college. But yeah, it was really, it was really quite forward thinking. So occasionally if I was caught out, I would actually, you know, go and use that, but there were staff toilets as well… And it was…the new member of staff was sort of saying, you know, it's really important that we also have that kind of thing in the in the ladies’ loos as well. And also sort of make it known that we can have like spare pairs of underwear and spare, like clothing and stuff, should anyone be caught out, which I thought was absolutely brilliant.” [#4]*

### Emotional support

Many of the women desired emotional support as they navigated through the mood swings and various other bodily changes they experienced. They expressed a desire to be supported while they learn to understand and navigate this new phase.

*“The most support I need…is the emotional support…I'm quite emotional. So…during my period, I enjoy emotional support. Where, uh…If I want my space, You give me my space. If I want you to help me with reading a book or…listening to me- just being that supportive. Helping me to cope with the difficult emotions that I'm experiencing. I just…need my- kids to…help me with that…if I need my space, you know you have to respect that as well, and then also a lot of support that I enjoy is on the product support…if one gets me…the specific pads, I- you know, and then for the heat pads for the cramps and all” [#31]*

Additionally, some said that the support of their husbands was important, as well as understanding from their children and friends. Some of the women stated that having friends or their mothers around to talk about their experiences also helped and provided comfort in knowing that what they were going through was normal.

*“I have support from friends, basically. And I think that's the primary support I have. Because I have friends that are my age and have experienced this before me. So that's where I get most of my support, and from family that have been moved passed this.” [#16]*

### Support groups

Another form of support some of the women desired were support groups – somewhere they could discuss their symptoms and experiences with other women going through the same thing. The ones who wanted this type of support described a desire for sisterhood and the comfort of knowing they are not alone on this journey.

*“And we can also have support groups. So, we talk about experiences and symptoms, and we know that we are not experiencing it alone.” [#13}*

*“So, I think that-that space. Lots of women's retreats and lots of oh, lots of circles for women. But having that* ***more*** *that I can go to as well, so I'm not running it. So, you can drop into that sisterhood and that collective support of other women coming together, nurturing and nourishing each other.” [#25]*

One woman described an online group that she had recently discovered and found helpful for a plethora of topics concerning perimenopause. She mentioned that she had originally sought the group for advice on digestion issues but discovered other helpful topics.

*“I’m not advertising, but I recently joined an online membership group which provides education, exercise, no subjects off topic, lots of nutrition advice. Medical input too. Harley street at Home - there are Facebook groups too which you can be part of without joined paid membership…For me, I came to it from digestive support, but the range of information and in particular, exercise options has been fabulous.” [#10]*

### Question 5: How could we improve education around menstruation and perimenopause?

When asked how education around menstruation and perimenopause could improve, they had a plethora of suggestions. Four themes were identified from their responses: normalising menstruation through conversation, education in schools, training healthcare professionals and spreading awareness through social networks. They also encouraged better, ongoing education in schools and workplaces. Additionally, some suggested utilising social networks to ensure those past school-age received education.

### Normalising menstruation through conversation

A common thread throughout the focus groups was the need to talk about menstruation and perimenopause. The women were adamant that speaking more openly about these topics would bring more awareness and normalisation to periods, ultimately dismantling stigma associated with menstruation. This included speaking to children at home and in school, colleagues, family members, friends – anyone, as long as there was someone to go to with questions and concerns.

***“*** *I think, first of all, it's talking…I think the more people come together and talk and share their knowledge and their experiences…because if you talk to other women, you suddenly realise that you're not different. You're not weird. It's not strange, what’s being gone through. And so, it removes that feeling of isolation.” [#25]*

*“The talking more - it's actually talking more. I make a big point of talking about periods in front of my teenage son and say, ‘you need to know this.’ I'm not hiding packets of tampons away that's in the bathroom, we need to know.” [#22]*

Though everyone agreed that talking is key to spreading awareness, one participant felt that, while effective, sole responsibility should not lie with women to spread awareness.

*“And when I went to the hairdressers about a month ago, I was sort of saying about being peri[menopausal] … And she said, ‘You know what, gosh.’ She said, ‘I’m gonna have a chat with my with my mum, because I think she needs to get on HRT* (hormone replacement therapy)*, I think…I'm pretty sure that this is what she's going through, and she thought she was depressed’ …that talking to other women that we…it…shouldn't have to be communicated in that way.” [#4]*

Some women shared how talking with others has helped them or their children feel more comfortable. Some noted that even though society has a long way to go, today’s generation has shown progress over past generations. A few explained that they tell their children about their periods and do not hide their menstrual products to normalise it.

*“And you know, my, my background is Asian and Muslim. So, you know, we don't discuss these things…we just suffer in silence. And there's just so much shame attached to the bleeding… if I could, I'd like to just lose the shame. But what I have noticed more recently is we're living in a different world now, and the thing is like, you know, even the fact that you can say the word menopause… I think for me, I think removing the shame, the stigma. and you know, why should we feel ashamed?” [#17]*

*“I try and talk to my boys about it about…using cloth things. And what I'm doing with it all. And they're like - my oldest is 18, is like, ‘Oh, that's disgusting.’ It's like, no, it's not. This is actually life. And this is real. And you know, trying to break down those stereotypes of ‘disgusting’.” [#25]*

### Education in schools

Almost all the women agreed better and continuous education in schools around menstruation is paramount. They stated that education should begin at an earlier age, as some girls get their periods earlier than they are being taught about it. One woman suggested teaching about menstruation at an earlier age, then introducing menopause a year or two later, to build on the concepts. A few of the women noted that giving the positives alongside the negatives, to empower girls and young women with the knowledge of their bodies.

*“I still think…that there needs to be, not just one-off things here and there, it needs to be something that's continuous and flows through school…” [#26]*

*“You say both the negative and the positive. They have to know how body works…when I was young, I thought at my age…I wouldn't be feeling all these things again, but then I'm still having them. So, I would say, both the negative and the positive should be, you know, made known to the teenage girls.” [#23]*

*“I think I think the education about perimenopause and the menopause needs to be started, you know, with school age children. So they understand what their par- what their mums are going through…but to do it in such a way that doesn't frighten them as well and…just thinking about ways in which it can be done in a positive way to empower women, and the men that are around them, to feel like they know what's happening and when to you know, seek, seek support, I think would be would be really, really helpful.” [#5]*

Many said boys should be educated about menstruation alongside girls, as they should be aware of what peers and family members are going through. In addition to teaching boys, the curriculum needs to be more than basic biological processes with lessons teaching girls how to take care of their bodies and hygiene.

*“I think they just teach [students] for the learning purpose, but they don't get to make it very holistic, so that the females get to know everything about menopause, how to take care of themselves, and male gender get to learn about how to be welcoming to females and not make them feel embarrassed when they have periods.” [#7]*

*“But I think the boys need to be taught about it as well as the girls. I know that that can be difficult in a teenage environment, but I think it's super important for them to learn what their friends and their siblings and their moms and their aunts are going through, and how, you know, not just in terms of menopause, but in menstruation generally…” [#19]*

*“…my sex ed was segregated by Gender and I think in Canada, I mean, fine. And I think like, that's ridiculous. Everybody needs to know it. Like I've to learn about the penis, you can learn about my uterus.” [#28]*

One woman thought schools should take it one step further and provide menstrual products for free to further tackle menstrual stigma and allow younger girls to understand that purchasing and using menstrual products does not have to be embarrassing.

*“I'm also thinking… if there's proper access to menstrual products, you know, given in schools…it's to make the teenage girls know that “Okay, these things are things that we need. These things are not…weird. They are things that are actually important for my hygiene. So, I should not be ashamed of going to the grocery shop to get these things, to the supermarkets to get the pads, the tampon, you know.” [#31]*

### Training healthcare professionals

Some of the women were advocates for making sure that healthcare professionals receive specific women’s health-related training. They desired GPs with more than basic knowledge of women’s bodies, more specialised knowledge of menstrual norms, the menopause transition, treatment options and individualised care so they could provide better care for women.

*“And I also think training healthcare providers, enhancing medical training programs to include comprehensive education on menopause and perimenopause could also go a long way to help, this will enable healthcare providers to better understand the symptoms and the treatment options, and then appropriate management strategy.” [#15]*

One woman said that training on women’s health issues, especially menopause, should be mandatory for all GPs wishing to treat women. She went on to say that if they opted out of the training, that they would be opting out of treating women. A few women agreed with this idea. Another woman agreed, stating that GPs need training to spot signs of perimenopause earlier on, as symptoms can begin prior to a woman reaching her mid-40s or beyond.

*“I think it should be compulsory for GPs to do all the training on the menopause. And if somebody wants to opt out of that, okay, well, you're also opting out of treating women then. You don't get to treat women if you haven't studied this, if you don't know the answers to some very basic and simple questions…I think it has to start with your health care provider. Has to. Because if if they don't know what they're talking about, then it's very hard to expect the teachers to be teaching about it because, you know, the teachers don't have a medical degree.” [#19]*

*“And I'd say like 10 years ago, I was obviously having symptoms. And…my GP said, ‘you're too young.’ Right? So, I'm still like, late 30s. I think there's this idea that your symptoms are going to hit you between your mid-40s and your mid-50s. And anything outside of that window, anything before mid-40s, it's not perimenopausal, it's something else. And I think that's the education, that knowing you could get symptoms from your mid-30s onwards. And anything that's slightly odd, you know, don't dismiss it as, you know, it could be perimenopause.” [#21]*

### Spreading awareness through social networks

Some of the women suggested that effort should be made to reach those who did not receive adequate menstrual education during school. They said that educating that group would help the women entering or experiencing perimenopause to better understand what their bodies are going through. Additionally, they said teaching those women would equip them with the knowledge necessary to speak to their children about perimenopause. Spreading awareness this way, they reasoned, would relieve some of the pressure from teachers. One woman said that educating as many as possible and meeting them where they are, would go a long way in giving people the ability to make choices that best align with their menstrual wants and needs.

*“I would like there to be an education of our generation, because we, a lot of us, have got children, a lot of us are going to be ones…spreading the word for younger generations. Otherwise, you're waiting for another 40 years. If you're teaching the kids [who] are at school now and putting the pressure on the education system, if you can give some tools that you're- you're multiplying that message…I've got the group, the generation that…is literally defining terms, let alone defining symptoms and giving people options and choice, and empowering them to look off, take their own choices, and I think it's about reaching a much wider group of people and the different levels. There are people that will read the books. There are people that will deal in communities. But then there's other people that just need it on the phones that will help them make their choices given whatever lifestyles they lead.” [#6]*

*“I wonder if it's mainly about getting somehow educating parents, because they're the ones that you know within the most, and I feel like they would have, you know just to sort of say, even if it's just to say, ‘look, things are going to change, come and talk to me.’” [#29]*

They also told the team that including men would be helpful and that men do want to be included in discussions. Bringing men into the conversation would help fight menstrual stigma by making it less of a taboo topic.

*“I think it'd be really nice to bring men more into the conversation, and I have spoken to men who have expressed that they want to be more part of the conversation, but they feel either frozen-out or they feel nervous about impinging on groups where women want to have a safe space…” [#26]*

Participants wanted to spread as much awareness and fact-based education as possible, as quickly as possible. While many agreed that progress has been made regarding curriculum and stigma, their responses showed that there is a long way to go.

### Treatments

When it came to treatments, many of the women expressed their desires to have options laid out in one trustworthy location, such as the NHS website. Some felt that it was too burdensome to scour the internet looking for answers, and not knowing if the source is reliable or not.

*“I cope well…More is about information like this is too much information, like it gets to be like too much…I can't take hormone-I become crazy if I take hormones…I have also breast cancer background. So, they said no HRT for me, so I might be worried like if it gets worse, which way? Where I will find the support?…That's why I need support to filter what's out there.” [#1]*

One woman suggested a website dedicated solely to perimenopause. She thought it could house information on perimenopause symptoms, treatments and other information all in one space. Additionally, she thought it could include a chat feature to connect the user with a GP, answer questions or book appointments.

*“There should also be a website where there'll be maybe a little bot on standby, or a GP that can always put it through whatever you want to know about it, or where you can book appointments online, just to know whatever you want to know about it.” [#9]*

It was suggested that this resource not only include HT or contraception options, but also more alternative, natural options as well. Many expressed a desire to avoid dependence on HT or other medications and wanted to know what alternative/natural treatments effectively alleviate symptoms.

*“The communication about herbs and nutrition, and the things that we can do that will really benefit because there are so many things that you can do to support your cycle that most people haven't got a clue about, and I think, having that in there is such an important thing, so that you've got other tools to turn to other than just the HRT. And things like that. It's like, well, actually, there's a whole breadth of knowledge that we could look at. And you know it's, I think you know the different foods as you get to go through different stages that will support your body as well or not support your body.” [#25]*

Further, though the team did not ask what treatments the women were using, many freely volunteered the information. Some women tried lifestyle changes, such as exercise and diet changes to relieve symptoms.

*“Some of the things that are really important are making more time for myself. So, more time to unwind, more time to go and do nice things- that self-care, like cold water swimming. That's been really helpful, you know. It's- it's those things that are going to kind of re-balance me you know. For me, nutrition and the supplement is really important. That's obviously part of my field. So really focusing in on that. Going to the gym and exercising. I've never liked the gym, and suddenly I love the gym, and pushing myself to do more makes me feel so much better. Swimming in the pool, you know, just- just having that time.” [#25]*

Others used natural herbs and supplements as their preferred options, although not all the remedies discussed are licensed as safe or proven treatment options. One woman explained that she had been using cannabidiol (CBD) oil as her way of dealing with PMS. She mentioned that she recommends it to friends who are experiencing similar symptoms.

*“I definitely noticed that PMT came back…PMT that had been under control for very long time, and it came back with a vengeance, and then it started to become more than a week…for a few months…I started to do my own head…now I find if I take CBD oil every day… to begin with, I would take it every day for the week before my period, and that would be fine, but now I have to take it every day of the month, and then I'm fine, and if I run out, or I forget to take it for a few days I can feel the irritability…” [#26]*

A few spoke to us about using HT as treatment. Some of the women using HT found it helpful, while others said that it made them feel terrible.

*“And thankfully, since I've been on HRT, most most of my symptoms, and especially the kind of awful sort of depression-like symptoms, which were just horrific, have pretty much gone. But there's been a couple of days when I have literally just cannot stop crying, or just, you know, I call it like, the cloud of sadness just descends on you, and you just feel shit, you just just feel absolutely terrible.” [#4]*

*“I tried HRT for a couple months; I can't handle my hormones. It made me bananas.” [#28]*

### The ‘Celebrity Effect’ and medicalisation

Additionally, many cited a menopause documentary as how they learned about perimenopause, with some saying it spurred them to visit their GP and seek treatment. Others expressed their frustration with the ‘Celebrity Effect’ that has led to HT being pushed as the main treatment option.

Some of the women felt grateful for the menopause documentary and for bringing awareness to perimenopause. A few said that after seeing the documentary, it prompted them to talk to friends or their GP.

*“As to myself and perimenopause I really had no idea this was coming...but the first symptoms, the brain fog and the weird sleeping and suddenly getting hot and cold it was like, "what is this?" I-sounds incredibly naive, but I really didn't see it coming until I started kind of experiencing things myself and then good old Davina on the TV, and then talking to friends and realising okay, right I need to get my head around some of this stuff.” [#22]*

Others who were familiar with the documentary felt that while it started a great awareness campaign, they felt it was used for pushing HT and selling products for menopause without knowing if those products were actually useful.

*“I mean, I know Davina has done great things recently and this and that, but I feel like that's all about HRT, HRT. And my husband's like, ‘you need to get on HRT.’ I'm like, No. Well, I said, I'll research it…But now it's like, the only information we have is HRT.” [#27]*

**Figure 1.**
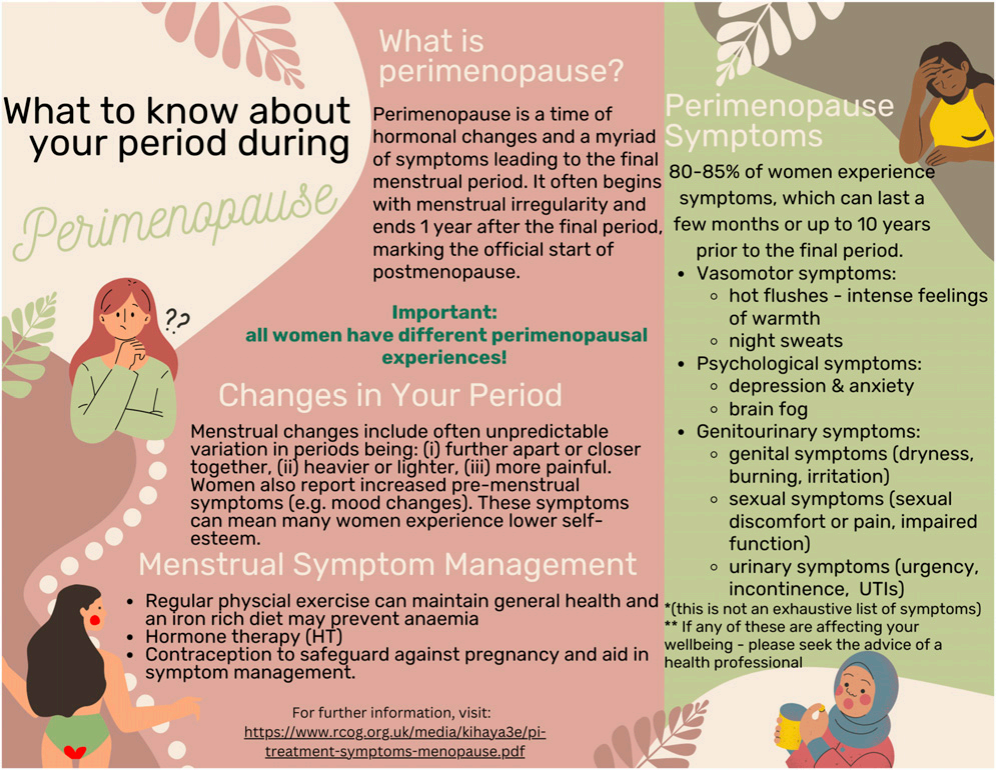
Perimenopause leaflet.

Figure 1 is an informational leaflet designed to provide perimenopausal women information with what kind of period-related changes to expect during perimenopause, based on the results found in the study.

## Discussion

We aimed to explore and analyse perimenopausal women’s feelings towards periods and their impact on wellbeing. Each participant expressed negative sentiments and significant impacts. Whether physical or emotional, perimenopausal women deal with a variety of changes that have a significant impact, an experience encountered by every woman in the study.

Additionally, the study aimed to identify how best to support women during perimenopause. Participants highlighted the importance of family, friends, workplace policies and community support groups. The women had many suggestions on how to improve support at the community and workplace levels through policies and social change.

We wanted to understand what improvements women thought should be made to menstrual and perimenopause education. Many lacked prior perimenopause knowledge from school,^19,40^ and suggested better incorporation into curriculum, specific healthcare training and employer understanding. Continuing to gather input from perimenopausal women is crucial for developing tools to enhance wellbeing.

### Period attitudes and wellbeing

In this study, most women reflected on initial menstrual experiences, which often shaped lifelong perceptions. Studies show many girls feel sad at menarche due to not being ready to ‘become a woman’.^41^ Previous research supports this, finding early distressing menstrual experiences led to negative attitudes later in life.^8^ Maravan and Chrisler found those who started their periods ‘early’ had more negative views.^9^ Many women in our study shared this sentiment, possibly due to feeling that menarche was unexpected and scary or that periods are embarrassing.^9^ The burden of periods was evident to many women in the study, with many feeling they had to endure physical and/or emotional pain.

While a few women found positives in the natural and cyclical aspects, negative associations persisted for all. Many were ready to celebrate their FMP. Women often feel shame and embarrassment associated with periods,^41^ a sentiment shared by many women. Even those who expressed appreciation for their cycles had negative feelings and experiences. Periods significantly and often adversely affect wellbeing, leading to negative menstrual attitudes.^42^

Beyond period intensity, voluntary and involuntary childlessness fuelled resentment towards periods. Voluntarily childless women reported wellbeing similar to parents with strong parent-child relationships, while involuntarily childless women were likely to report regret, grief and feeling ostracised.^43,44^ Many of the women who brought up their childlessness felt their periods were a waste of time, especially women who reported particularly troublesome periods. While mentioned repeatedly, there is not extensive research on menstrual attitudes of voluntarily childless women. Further research is needed, as attitudes and feelings of voluntarily childless women hold valuable insights.

### Menstrual changes due to perimenopause

Most women experienced menstrual variation in perimenopause, which ranged from lighter or heavier, longer or shorter periods, or longer or shorter cycles. The literature often glosses over menstrual changes, with variability being simply ‘persistent irregularity of the menstrual cycle’.^45^ During early perimenopause, women may note missed period(s) or variation in menstrual cycle.^5^ Santoro noted that cycle irregularity can be quite intense and becomes more irregular as a woman progresses further into the menopausal transition,^5^ with short cycle intervals more common during early perimenopause, and longer cycle intervals occurring later on.^46^

Many participants spoke of period unpredictability, from cycle length to period duration and bleeding patterns, which significantly disrupted their lives. The literature lacks comprehensive exploration of perimenopausal women’s lived experiences of such disruptions, though the FIGO system indicates that adolescents and women in their 50s experience more variation.^10^ This research addresses this gap by delving into women’s feelings about period changes and impacts.

Amid perimenopausal changes, significant period variations often disrupt normal functioning, causing distress, and confusion leading to feeling scared and uncertain.^19^ The combination of unpredictable cycles and emotional changes made perimenopause distressing for many.^47^ While many women in the study were familiar with menopause and were expecting changes, some had not heard of perimenopause. Regardless of expectations, many were caught off guard by the onset of symptoms. Some took time to realise symptoms were due to perimenopause. This mirrors earlier research, which found some women live with symptoms for a while before properly identifying them.^19^

### Impact on wellbeing

Reproductive health significantly affects women’s overall wellbeing throughout life. However, adverse symptoms often go unreported and overlooked due to Normalisation.^39^ Midlife is often a transformative phase involving bodily changes, fertility loss, children leaving home, caring for and losing elderly parents. These changes often lead to reduced social connections and QoL, and is sometimes a period of crisis for women.^45^

Despite perimenopause being a natural event, the unclear timing of perimenopausal symptoms causes unnecessary distress,^47^ and trouble coping with daily life.^45^ Women with higher period pain and menstrual duration are more likely to find periods bothersome.^48^ Further, HMB during perimenopause is shown to diminish QoL.^48,49^ Onset of adverse mood symptoms negatively affect those experiencing them, suggesting the value of anticipatory education.^47^

Perception of ageing and awareness of fertility loss can be detrimental to self-image and self-esteem.^45^ More perimenopausal symptoms correlate with reduced self-esteem and confidence.^50,51^

### Support

Many women emphasised the crucial role of having a support system, from family, friends and spouses to community support groups. Accessibility to online or in-person groups was vital for those with limited resources. For those with office jobs, workplaces were identified as a key area needing support. 30%–40% of women reporting perimenopausal symptoms reduce performance and social perceptions of desirability.^45^ They desired knowledgeable managers and a designated point of contact to address menstrual issues and provide necessary support, aligning with BSI’s ‘Menstruation, menstrual health and menopause in the workplace – guide’.^38^ Guidelines include the need to address physical and environmental workplace aspects, implementing flexible work arrangements, and fostering inclusive environments that support menstrual and perimenopausal health.^38^

In July 2023, after this study’s focus group session wrapped up, the Employment Relations (Flexible Working) Act 2023 was enacted,^52^ granting ‘day-one’ flexible work request rights,^53^ potentially aiding perimenopausal women in gaining more work-from-home flexibility.

The study revealed instances of insufficient healthcare support, particularly visits pertaining to women’s health-related issues where many of women felt dismissed or brushed aside, mirroring medical gaslighting seen in other research.^19^ Women recounted dismissive attitudes from healthcare professionals, often leading to later ‘discovered’ medical issues. Some women who seek care end up being wrongly prescribed anxiety or depression medications for their symptoms.^19^ For women with AUB, it is estimated that up to half of those affected do not bother seeking medical care,^10^ a trend supported by this study’s findings.

### Period and perimenopause education

Few participants were prepared for menarche or perimenopause. Most thought schools lacked comprehensive education on the lived experiences of periods and perimenopause and how best to manage them, other than some basic biology – which many felt was given too late to be beneficial. One study found women in their 50s had received little menopause education and desired more education around symptoms and treatment.^54^ The Women’s Health Strategy for England (August 2022) highlighted menopause as a priority, resonating with women’s desires for more information.^55^

Current UK school curricula introduce periods, but not holistically, and is perceived as being introduced too late. A study indicated 75% of women under 40 believed menopause should be taught in schools.^40^ Studies show including boys in menstrual education normalises menstruation and conveys an understanding of what peers and family members are going through.^19,40^ The UK’s updated secondary school RSHE curriculum (introduced in 2019) indicates that students should understand reproductive health ‘and menopause’^56^ – a phrase that reads as though menopause was tacked on as an afterthought. The Women’s Health Strategy aims to educate girls and boys about menopause from an early age, as it acknowledges the societal impact.^55^ Including menopause in the RSHE was crucial and will help future generations, if thoroughly taught. Teaching men and boys about menstruation and perimenopause to fight stigma and gain understanding was upheld by the adamant women of this study. This paper is part of a study where focus groups will be conducted with a wider range of women. This has already been repeated in schools with year 10 girls (14–15* *year olds) where they were asked 3 of the 5 questions in this study that did not relate to perimenopause. The girls called for boys to be included, for more education with appropriate teachers, improvements in school toilets, and for teachers to be taught how to support those having periods (Brooks, Maybin and Harper, in preparation).

Inadequate education leads healthcare professionals to lack the ability to recognise perimenopause and menstrual issues,^19,54^ raising concerns among women. Knowledge gaps for diagnosis and treatment arise from insufficient education and clinical training.^57^ A staggering 41% of UK universities exclude menopause from the curricula.^58^ Women believe in specialised training for GPs in women’s health and perimenopause, encompassing symptom recognition and proper treatment. The BMS and Practice of Menopause Care have a training program to ensure healthcare professionals have comprehensive, evidence-based educational resources on menopause care.^59^

### Limitations

Limitations in employing social media platforms to promote the study must be noted, including the potential introduction of selection bias. Moreover, it is plausible that participants who undergo particularly distressing perimenopausal experiences might exhibit a heightened propensity to both follow the selected social media platforms and enrol in the study, thereby potentially skewing the study towards a prevalence of accounts recounting adverse challenges.

Purposive sampling was employed to ensure a relatively diverse group of women. However, at the start of this project, we found an issue that has been discovered in other studies. People who did not fit the inclusion criteria were enroling in the study giving false demographics on the consent form to earn the £25 voucher. This was picked up quite early and participants were informed prior to the zoom meeting that they would have to turn on their camera, which caused some participants to withdraw.

However, even acknowledging this selection bias, we feel it is important to hear these women’s stories and take their needs into account when developing policy and guidelines.

### Recommendations

We recommend that menstruation and menopause are holistically taught in schools, early, consistently and continuously to create deeper understanding and breakdown stigma. Helping women and men prepare for changes that accompany perimenopause will allow women to transition into this period without fear and confusion and help men empathise with what they are going through. Work is being done in this area by the International Reproductive Health Education Collaboration who have been working on a teacher’s guide to reproductive health.^19^

UCL have launched the UK Menopause Education and Support Programme, in conjunction with Wellbeing of Women, BMS, Royal College of Obstetrics and Gynaecology (RCOG) and Sophia Forum. The program will be similar to antenatal courses offered to pregnant women, but will enable perimenopausal women to be educated and get peer support.^60^ It will prioritise evidence-based education, connection with other women with similar experiences, and most importantly, will keep women’s voices at the heart of the design and implementation of the program.

Finally, we recommend that healthcare professionals receive adequate training on women’s health, especially perimenopause such as the British Menopause Society (www.thebms.org.uk) and the Royal College of Obstetrics and Gynaecology. Teaching on menstruation and menopause should be maintained in undergraduate medical curriculums and consideration should be given to mandatory post-graduate courses for general practitioners. This is crucial to help women navigate perimenopausal symptoms and changes. Continuing professional education for health professions would facilitate better management for women seeking care, and potentially reduce the number of visits from women who would otherwise be continuously seeking answers.

## Conclusion

This study offers valuable insight into the impact of periods on perimenopausal women’s wellbeing, and the persistent influence of attitudes established during menarche then carried on through life. These negative menstrual experiences are often due to lack of education about what is and is not normal, and when to seek medical care that leads to diminished QoL. However, when women do seek care, it appears that medical professions are ill-equipped to address period-related issues, particularly perimenopause. Early, inclusive and comprehensive menstrual education is vital for everyone, alongside specialised women’s health training for healthcare professionals. Empowering women with knowledge aids self-advocacy and informed treatment choices. Finally, accessible support is essential for each woman’s perimenopause journey. Much progress has been made in recent years, but continued momentum is crucial to keep building resources for support and education.
